# Recent Advances in Fluorescence Imaging of Pulmonary Fibrosis in Animal Models

**DOI:** 10.3389/fmolb.2021.773162

**Published:** 2021-11-02

**Authors:** Zongwei Liu, Xiaofang Tang, Zongling Zhu, Xunxun Ma, Wenjuan Zhou, Weijiang Guan

**Affiliations:** ^1^ Department of Respiratory Medicine, Lianyungang Hospital of Traditional Chinese Medicine (TCM), Affiliated Hospital of Nanjing University of Chinese Medicine, Lianyungang, China; ^2^ Green Catalysis Center, College of Chemistry, Zhengzhou University, Zhengzhou, China; ^3^ Department of Respiratory Medicine, Pukou District Hospital of Chinese Medicine, Pukou Branch of Nanjing Hospital of Chinese Medicine, Affiliated to Nanjing University of Chinese Medicine, Nanjing, China; ^4^ Department of Chemistry, Capital Normal University, Beijing, China; ^5^ State Key Laboratory of Chemical Resource Engineering, College of Chemistry, Beijing University of Chemical Technology, Beijing, China

**Keywords:** pulmonary fibrosis, imaging, nanomaterial, biomarkers, near-infrared

## Abstract

Pulmonary fibrosis (PF) is a lung disease that may cause impaired gas exchange and respiratory failure while being difficult to treat. Rapid, sensitive, and accurate detection of lung tissue and cell changes is essential for the effective diagnosis and treatment of PF. Currently, the commonly-used high-resolution computed tomography (HRCT) imaging has been challenging to distinguish early PF from other pathological processes in the lung structure. Magnetic resonance imaging (MRI) using hyperpolarized gases is hampered by the higher cost to become a routine diagnostic tool. As a result, the development of new PF imaging technologies may be a promising solution. Here, we summarize and discuss recent advances in fluorescence imaging as a talented optical technique for the diagnosis and evaluation of PF, including collagen imaging, oxidative stress, inflammation, and PF-related biomarkers. The design strategies of the probes for fluorescence imaging (including multimodal imaging) of PF are briefly described, which can provide new ideas for the future PF-related imaging research. It is hoped that this review will promote the translation of fluorescence imaging into a clinically usable assay in PF.

## Introduction

As a chronically progressive disease, pulmonary fibrosis (PF) features activation of myofibroblasts and subsequent deposition of large amounts of extracellular matrix, leading to the impaired gas exchange and the increased risk of respiratory failure (Richeldi et al., 2017; Ni et al., 2018; Hayward et al., 2021). In particular, PF is considered a risk factor for the severe evolution of coronavirus disease 2019, which is rapidly spreading and escalating into a global pandemic affecting the health of billions of people (Crisan-Dabija et al., 2020; George et al., 2020). Given that lung transplantation is the only effective treatment for PF to date (Juillerat-Jeanneret et al., 2018), the early diagnosis of PF is critical to help reduce patient morbidity and improve patient survival.

Currently, PF is primarily diagnosed by high-resolution computed tomography (HRCT) imaging (Wu et al., 2019; Mento et al., 2020). However, lung structures in early pulmonary fibrosis may not be deformed, making it difficult to distinguish fibrosis from other pathological processes (Désogère et al., 2017). HRCT also faces challenges in detecting idiopathic pulmonary fibrosis (IPF) because of its atypical appearance on HRCT images (Liu et al., 2017; Barratt et al., 2018; Ley and Ley-Zaporozhan, 2020). A practical alternative is to use the magnetic resonance imaging (MRI). Although the presence of an air-tissue interface usually results in a low proton density and poor magnetic susceptibility environment in the lung (Mammarappallil et al., 2019), MRI using hyperpolarized gases (e.g., helium or xenon) can reveal subtle changes in lung microstructure (Lonzetti et al., 2019). Unfortunately, the high cost constrains it from becoming a routine diagnostic tool. Therefore, the development of cost-effective and highly-sensitive imaging techniques for the early diagnosis of PF is necessary to assess the status of the lung and to guide patients toward the appropriate clinical pathway.

Fluorescence imaging is based on the detection of electromagnetic radiation in the ultraviolet–visible–near-infrared (UV–Vis–NIR) range emitted by fluorophores (Ximendes et al., 2021). Thanks to the advantages of high sensitivity, tunable specificity, controllable cost, acceptable biosafety, and noninvasiveness (Fatahi et al., 2020; Guan et al., 2020; Niu et al., 2020; Guo et al., 2021; He et al., 2021), fluorescence imaging has been applied to visualize vascular system (Ma et al., 2018; Zheng et al., 2019), lymphatic system (Nakajima et al., 2018), various tumor tissues (Bai et al., 2020; Bai et al., 2021), and real-time blood flow (Hong et al., 2014; Wan et al., 2018; Cai et al., 2020). In addition, fluorescence imaging shows great potential in facilitating the development of precise therapeutic strategies for diseases, including imaging-guided cell therapy, drug delivery/release monitoring, and photodynamic therapy (Li et al., 2020; Li et al., 2019a; Liu et al., 2019; Han et al., 2021; Xu et al., 2021). It is clear that fluorescence imaging can play an important role in the diagnosis, treatment, and rehabilitation of PF. Herein, we summarize the knowledge of PF-related fluorescence imaging in terms of collagen imaging, oxidative stress and inflammation imaging, PF-related biomarker imaging, and imaging-guided PF therapy. The properties and drawbacks of relevant fluorescent probes, as well as possible solutions, are briefly discussed. By reviewing these recent advances in PF-related fluorescence imaging, we hope to contribute to potential protocols and ideas for future PF-related imaging studies.

## Imaging of Collagen

**FIGURE 1:**
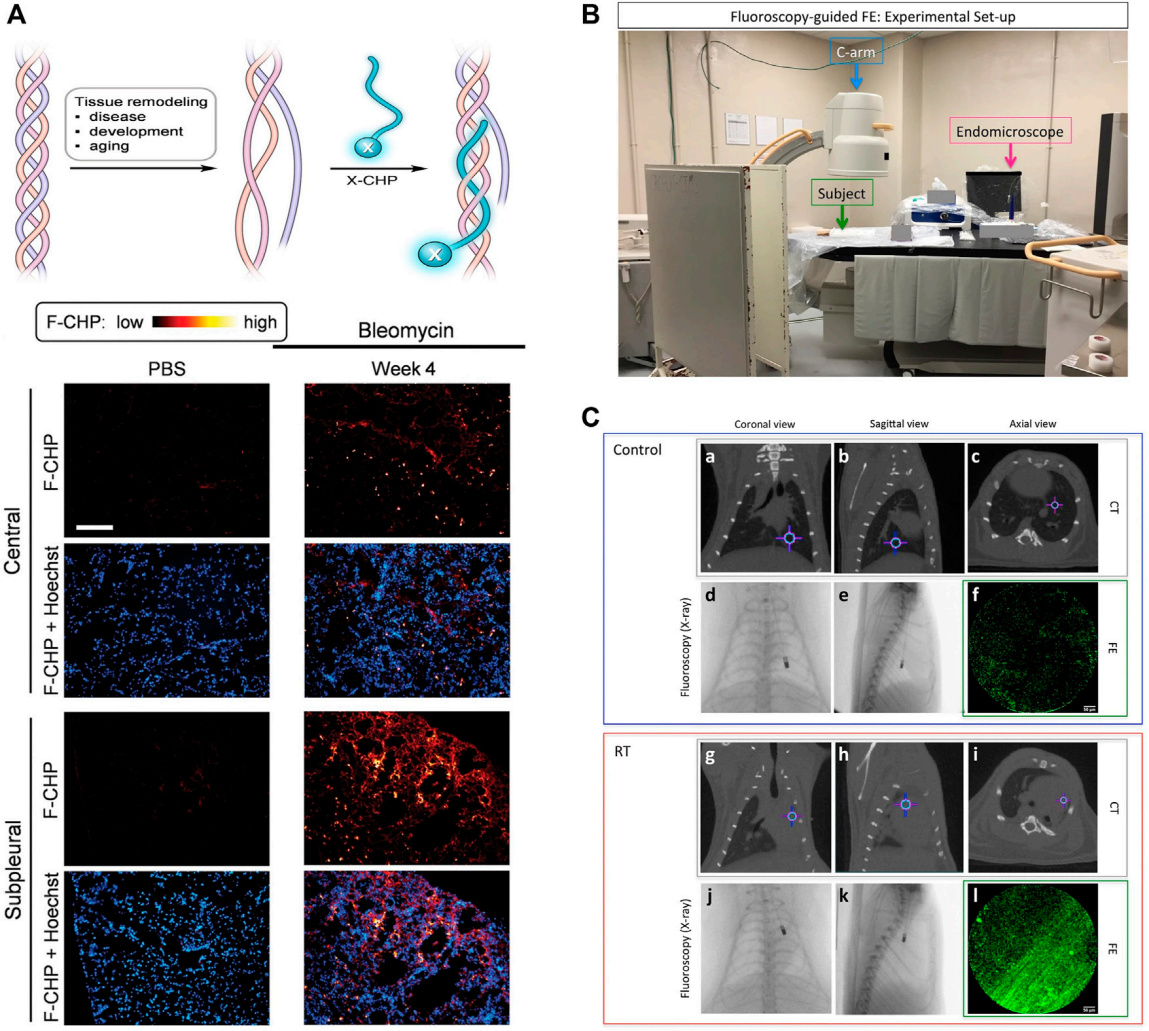
**(A)** Schematic of a CHP strand (labeled with X) hybridizing to denatured collagen chains and forming a collagen triple helix and representative fluorescence micrographs of the center and subpleural area of frozen lung sections, which were obtained from bleomycin mice and control mice. **(B)** Experimental set-up. **(C)** Representative CT (a-c and g-i, gray box) and FE (f and l, green box) images of control (a-f, blue box) and RT (g-l, red box) at the corresponding endomicroscope position determined with the fluoroscopy images (d, e and j, k, respectively). Crosshair indicates the position of the endomicroscope tip. Adapted and modified with permission from ref (Hwang et al., 2017) (Copyright 2017 American Chemical Society) and ref (Perez et al., 2017) (Copyright 2017; published by Springer Nature).

Collagen is the main structural component of the extracellular matrix in mammalian tissues. The deposition of collagen is a common feature of the PF patients (Snijder et al., 2019; Sorushanova et al., 2019; Yombo et al., 2021). Currently, collagen staining, collagen antibodies, and imaging systems (second harmonic generation and transmission electron microscopy) face the challenge of distinguishing structural changes between intact collagen and degraded collagen at the molecular level (Nielsen et al., 1998; Fligiel et al., 2003; Matteini et al., 2012; Hwang et al., 2017a). Inspired by the unique triple-helical structure of collagen, a collagen hybrid peptide (CHP) was developed to have specific binding to denatured collagen chains but negligible binding to intact collagen (Hwang et al., 2017b). By linking 5-carboxyfluorescein (green-fluorescent dye) on CHP, *in situ* fibrotic changes in frozen sections of lung tissues from bleomycin-treated animal models were observed by fluorescence microscopy. As shown in Figure 1A, bright spots indicated the damaged collagens bound to CHP *in vivo*. The signals in the central and subpleural areas increased over time, while the number of bright spots in the subpleural area increased more significantly, providing direct evidence for the structural abnormality of collagens in fibrotic tissues.

Compared to single-modality imaging, multi-modality imaging tends to have higher sensitivity and specificity by combining the advantages of various imaging techniques, allowing for a more comprehensive evaluation of PF-related targets (Perez et al., 2017; Ruscitti et al., 2018). For example, CT imaging could provide macroscopic anatomical information in clinical diagnosis, while fluorescence endomicroscopy (FE) using targeted probes enabled the observation of microscopic fine structures. A collagen-targeted fluorescent probe was conveniently designed by replacing the signal unit of the reported MRI probe with fluorescein isothiocyanate (FITC). The obtained Ac-Lys (Ac)-Trp-His-[*Cys-Thr-Thr-K(FITC)-Phe-Pro-His-His-Tyr-Cys]-Leu-Tyr-Bip-Amide showed satisfactory binding affinity to collagen in a plate binding assay (Perez et al., 2017). Accordingly, a correlation between lung density on CT images and collagen fiber structures on FE images was observed at any given corresponding endomicroscopy location (Figures 1B,C). In addition, another dual-modality imaging that can evaluate the progression of bleomycin-induced PF in mice was constructed by changing fluorescent probes (Ruscitti et al., 2018). Fluorescence molecular tomography (FMT) using a matrix metalloproteinase fluorescent probe could reveal quantitative information about the expression of IPF protein *in vivo* and *in vitro*, while micro-CT imaging reflected the pathological and therapeutic status of lung parenchyma. There is no doubt that PF-related multi-modality imaging has great potential and deserves more attention and effort.

## Imaging of Oxidative Stress and Inflammation

PF patients are susceptible to high levels of oxidative stress because the lung is directly involved in respiration (Cheresh et al., 2013; He et al., 2019). Oxidative stress was positively correlated with the reactive oxygen species (ROS) levels (Schieber and Chandel, 2014; Song et al., 2021). Moreover, early inflammation in PF may also increase the ROS levels (Wang et al., 2020). Therefore, ROS-responsive fluorescent probes hold promise for the early diagnosis of PF.

Mitochondria are the main source of cellular hydrogen peroxide (H_2_O_2_) (Wei et al., 2021). Mitochondrial-targeted fluorescent probes are more likely potential strategies for further understanding of the mechanism of the disease occurrence and progression, as well as early diagnosis and treatment of the disease. A simple and effective method is to introduce mitochondrial-targeted groups (triphenylphosphonium) into the fluorescent scaffold (Song et al., 2021). Selective detection of mitochondrial H_2_O_2_ was realized by the combination of the azo-BODIPY (NIR-fluorescent dye), 4-(bromomethyl) phenylboronic acid pinacol ester (H_2_O_2_ response unit), and triphenylphosphonium cation (mitochondrial-targeted group) in one molecular probe (Mito-Bor). It showed little fluorescence because of photoinduced electron transfer (PET) between azo-BODIPY and 4-(bromomethyl) phenylboronic acid pinacol ester. In the presence of H_2_O_2_, the NIR fluorescence (∼730 nm) of Mito-Bor was turned on due to the inhibition of PET. Subsequently, changes in H_2_O_2_ concentration during fibrosis were successfully visualized by *in vitro* and *in vivo* fluorescence imaging, which indicated the levels of oxidative stress in fibroblasts.

**FIGURE 2:**
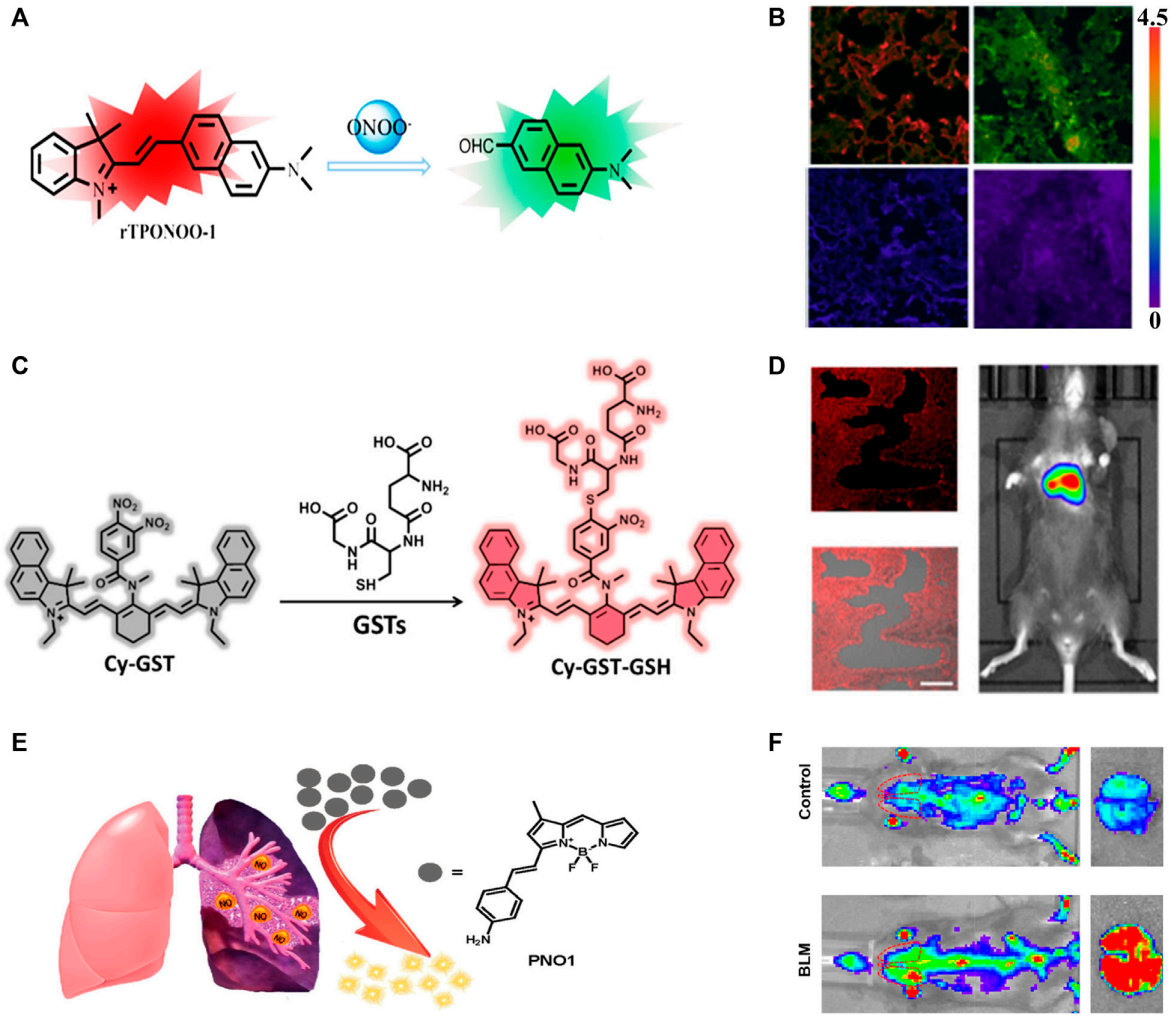
**(A)** Detection mechanism of probe rTPONOO-1 to ONOO^−^. **(B)** Imaging of ONOO^−^ levels from healthy lung slices to lung inflammation and pulmonary fibrosis. **(C)** Proposed Detection Mechanism of the Probe Cy-GST against GSTs. **(D)** The GST concentration of PF mice at 28 days of bleomycin stimulation, and the fluorescence imaging and merged imaging of fluorescence and bright-field imaging of lung slices by treatment with Cy-GST. **(E)** Schematic illustration of the sensitive detection of NO by the fluorescent probe PNO1. **(F)** Representative images of control or bleomycin mice, and the lungs isolated from indicated groups. Adapted and modified with permission from ref (He et al., 2019) (Copyright 2019 American Chemical Society), ref (Zhan et al., 2019) (Copyright 2019 American Chemical Society) and ref (Dong et al., 2020) (Copyright 2019 American Chemical Society).

The peroxynitrite (ONOO^−^) response groups can also be reduced fluorescent dyes that show no or weak fluorescence, but exhibit intense emission after oxidation by ONOO^−^ (Wang et al., 2018). For example, oxazine can convert to a NIR-fluorescent dye (SiO3) by replacing the bridging O-atom in oxazine with a Si-atom (Wang et al., 2020). The fluorescence intensity of the reduced species (HSiO3) could be enhanced 208-fold or 216-fold after treatment with hypochlorite (ClO^−^) or ONOO^−^ to form SiO3. The off-on fluorescent switch performed well in fluorescence imaging of HClO/ONOO^−^ in IPF mice. In comparison with single-photon fluorescent probes, two-photon fluorescent probes usually have higher spatial resolution and imaging depth. A new two-photon NIR-fluorescent probe, rTPONOO-1, was obtained through direct condensation of acedan (two-photon fluorescent dye) and an indolium derivative (Zhan et al., 2019). Its C=C bond could be selectively cleaved by ONOO^−^, causing a blue shift of the emission wavelength from 718 to 535 nm (Figure 2A). Two-photon imaging in frozen lung sections exhibited high resolution at penetration depth up to 110 μm. Moreover, the fluorescence ratio changes of rTPONOO-1 in bleomycin-treated mice showed a good linear relationship with the concentrations of bleomycin (Figure 2B), providing the opportunity for the early prediction of PF progression.

Compared to the emission from the NIR-I (680–900 nm) window, the NIR-II (900–2000 nm) emission has higher penetration depth and less light scattering to facilitate better imaging (Wang et al., 2019a; Zhao et al., 2019; Liang et al., 2021). For example, efficient 1,060 nm emission could be achieved using glutathione (GSH)-modified lanthanide-based nanoprobes (Zhao et al., 2019). Higher signal-to-noise ratios were achieved by the generation of cross-linked nanoprobes that accumulated at ROS-producing sites (e.g., inflammatory regions), as a result of ROS-induced cross-linking between sulfhydryl groups in GSH. On the other hand, different from the absolute intensity mode, the absorption competition-induced emission between lanthanide-based nanoprobes and Cy7.5 fluorophores under dual-wavelength (808 and 980 nm) excitation could show ratiometric response to HClO (Wang et al., 2019a). The high-resolution ratiometric fluorescence imaging at 1,550 nm had a penetration depth of 3.5 mm in the scattering tissue phantom. Through the incorporation of phenyl borate group and benzothiopyrylium cyanines skeleton (Li et al., 2019b), a new turn-on fluorescent probe for ONOO^−^ (IRBTP-B) was constructed. The dioxaborolane group in the probe IRBTP-B was shown to be oxidized by ONOO^−^ and produce a fluorophore with entire conjugation, which revealed a NIR-II fluorescence emission. Tissue phantom study confirmed that reliable signal was obtained at a penetration depth of up to 5 mm.

In contrast to ROS, oxidative stress was negatively correlated with the levels of antioxidants (Pisoschi and Pop, 2015; Liu et al., 2018). GSH is a typical intracellular antioxidant and is found in relatively low concentrations in the lungs of PF patients (Wang et al., 2019b; He et al., 2020). Considering that cysteine is a raw material for GSH synthesis, cysteine levels are expected to be an indicator for the diagnosis of PF (Wang et al., 2019b). Photoacoustic and fluorescent dual-modality imaging probe CCYS consisting of a hemianthocyanine NIR-fluorescent dye and an acrylate response group was used for *in situ* monitoring of cysteine concentrations in PF mice. Higher cysteine concentrations were found in PF mice than in healthy mice, suggesting that low concentrations of GSH was not caused by low concentrations of cysteine. Further comparing the photoacoustic and fluorescence signals of CCYS in PF, pneumonia, and healthy mice, only PF mice showed a significant response, which provided a potential tool to differentiate PF from pneumonia. On the other hand, glutathione S-transferase (GST) and glutamyl transpeptidase (GGT) catalyze the nucleophilic addition of GSH to oxidative stress products and are thus potential indicators for monitoring PF progression (He et al., 2019; He et al., 2020). Two NIR fluorescent probes Cy-GST and Cy-GGT were obtained through the introducing of 3,4-dinitrobenzoic acid group (GST response unit, Figure 2C) and γ-glutamyl amide group (GGT response unit) to the cyanine (NIR-fluorescent dye). The PET-induced fluorescence quenching that occurred in Cy-GST and Cy-GGT could be selectively interrupted in the presence of GST and GGT. The specific imaging of GST and GGT concentrations in PF cells and mouse models demonstrated that higher levels of oxidative stress led to the increase of GST (Figure 2D) and GGT.

## Imaging of Pulmonary Fibrosis-Related Biomarkers

Cyclooxygenase-2 (COX-2) is an inducible enzyme closely related to PF (Robertson et al., 2012; Feng et al., 2019). A NIR-fluorescent probe Cy-COX, formed by covalently linking indomethacin (response group) and cyanine (NIR-fluorescent dye) with hexanediamine, has been successfully used to evaluate and image the level of COX-2 in organisms (Wang et al., 2021). The hydrophobic cavity of the COX-2 homodimer could be occupied by indomethacin, and Cy-COX adopted an unfolded conformation to suppress the PET effect with turn-on fluorescence. The imaging results of the PF mouse model showed that COX-2 played a role in alveolar cell inflammation and early PF through high expression.

Small metabolites are considered as sensitive markers for disease diagnosis (Wishart, 2016; Si and Lee, 2020). For example, the increase of dimethylarginine dimethylaminohydrolases (DDAHs) and inducible nitric oxide synthase (iNOS) could cause the up-regulation of nitric oxide (NO) expression in patients with IPF (Dong et al., 2020). The fluorescence of the probe PNO1 was significantly increased after treatment with NO. Its excellent sensitivity to NO enabled high-contrast imaging in diseased primary cells, tissues, living mice with bleomycin-induced fibrosis, and clinical PF tissues. Compared to the control group, the fluorescence intensity of PNO1 in the lungs of PF-affected mice was about 6 times higher, reflecting the ability of the probe PNO1 to sense local NO changes in the microenvironment (Figures 2E,F), and has a complementary effect on large-scale anti-PF drug screening. The properties of the above PF-related probes are briefly summarized in Table 1. It can be seen that most probes emit light in the NIR region, which can be used for deep tissue imaging. To further increase the penetration depth, NIR-II probes with longer emission wavelength are emerging, and promising for *in vivo* and *in situ* diagnosis of PF.

**TABLE 1:** Properties of PF-related fluorescent probes.

Probe	Target	Emission wavelength	References
Mito-Bor	H_2_O_2_	730 nm	Song et al. (2021)
HSiO3	HClO/ONOO^−^	760 nm	Wang et al. (2020)
rTPONOO-1	ONOO^−^	718/535 nm	Zhan et al. (2019)
Compound 1	H_2_O_2_	667 nm	Liang et al. (2021)
DCNP@GSH	H_2_O_2_	1,060 nm	Zhao et al. (2019)
DCNP@Cy7.5	HClO	1,550 nm	Wang et al. (2019a)
IRBTP-B	ONOO^−^	950 nm	Li et al. (2019b)
CCYS	Cys	710 nm	Wang et al. (2019b)
Cy-GST	GST	810 nm	He et al. (2019)
Cy-GGT	GGT	780 nm	He et al. (2020)
Cy-COX	COX-2	770 nm	Wang et al. (2021)
PNO1	NO	559 nm	Dong et al. (2020)

## Imaging-Guided Therapy

Fluorescence imaging can be a useful technique for guiding PF therapy in addition to the detection of PF-related collagen, oxidative stress and inflammation, and biomarkers. In response to the abnormal expression of biomarkers in the process of PF, different treatments have been proposed to help ease symptoms. The chemical structure of fluorofenidone (AKF) is similar to that of the anti-fibrotic drug pirfenidone, which can inhibit the growth of fibroblasts and the synthesis of collagen. Spermidine (Spd)-modified poly (lactic-co-glycolic acid) (PLGA) nanoparticles (NPs) were used as an AKF carrier to improve the anti-fibrosis effect (Tang et al., 2017). Coumarin-6 and 1,1′-dioctadecyl-3,3,3′,3′-tetramethyl indotricarbocyanine iodide (DiR) fluorophores were encapsulated in Spd-AKF-PLGA NPs for *in vivo* fluorescence imaging to give the distribution information of Spd-AKF-PLGA NPs during the therapy. Through the specific recognition by lung epithelial cells and accumulate in lung tissues, Spd-AKF-PLGA NPs reduced the degree of fibrosis and alveolar destruction in paraquat-induced animal models. Some ROS-sensitive fluorophore, such as nonfluorescent dihydrorhodamine-123, which can be rapidly oxidized by ROS to the strongly fluorescent rhodamine 123, allowed determination of the ROS level using fluorescence changes (Liu et al., 2018). When antioxidants (Liu et al., 2018) or ROS scavengers (Zhou et al., 2018) show great potential to balance oxidative stress in PF, inhibition of ROS production in treated cells can be observed by fluorescence imaging.

Stem cells are a type of unlimited self-renewaling cells that can migrate to injured tissues and differentiate into target cells after transplantation to exert their therapeutic potential (Chen et al., 2018). In clinical trials based on stem cell therapy, bone marrow mesenchymal stem cells (BMSCs) are often used to study the treatment of PF. Transplanted BMSCs exerted anti-inflammatory and anti-fibrotic effects by down-regulating the expression of pro-inflammatory cytokines and chemokines released by macrophage activation (Huang et al., 2020). Different types of NIR fluorescent NPs (such as quantum dots, rare-earth NPs, and organic fluorescent NPs) have been used for *in vivo* tracking of transplanted stem cells (Chen et al., 2018). It was confirmed that BMSCs labeled with AA@ICG@PLL NPs could be effectively transplanted into SiO_2_−induced PF mice, and then monitored by CT/NIR fluorescence dual-modality imaging for up to 21 days (Huang et al., 2020). Additionally, BMSCs were observed to migrate to damaged lung tissue when DiR-labeled BMSCs were transplanted into a SiO_2_−induced PF rat model (Li et al., 2018). Optical micrographs also indicated that injection of BMSCs did reduce SiO_2_−induced collagen deposition and the number of nodules in lung tissue, while causing up-regulation of the expression of alveolar epithelial markers. Thus, image-guided therapy provides a useful tool for studying the mechanisms of PF and offers a bright future for the treatment of PF patients.

## Conclusion and Outlook

On the basis of the pathological process of PF, this review covers the fluorescence imaging studies used in the last years for PF diagnosis and to assess the effect of relevant drug treatments. Currently, there are no other simple, rapid and effective diagnostic tools when HRCT or surgical lung biopsy is not possible, and the identification of biomarkers (*e.g.*, ROS, inducible enzymes, and small metabolites) would be of great help to perform the early diagnosis of PF. The use of specific fluorescent probes to label PF-associated biomarkers allows for real-time detection of fluctuating levels of these biomarkers, providing visual evidence of their abnormal expression *in vivo* and/or *in vitro*. Meanwhile, probes with NIR fluorescence emission can reduce background interference and provide high-precision imaging of deep tissues. On the other hand, multimodal imaging that combines fluorescence imaging and other imaging techniques shows great potential for generating more comprehensive information in PF detection and treatment response.

Despite some advances in fluorescence imaging for PF diagnosis, there are still some unresolved challenges. For example, ROS levels may be associated with other diseases and corresponding inflammation, causing the results of imaging and measuring ROS to be unspecific to PF disease. The translation of preclinical studies and *in vitro* tests to the clinic requires safe fluorophores and standard imaging protocols. A feasible solution is to select a suitable fluorophore among the existing Food and Drug Administration (FDA)-approved medicines as the fluorescent parent and to make reasonable modifications. Current fluorescence imaging systems also need to be further improved in order to be suitable for clinical applications. In addition, multimodal imaging may be able to accelerate the clinical translation of fluorescence imaging by using existing fluorescent probes in conjunction with other FDA-approved imaging agents.
